# Supplementary material for: Deletion of ER-retention motif on SARS-CoV-2 spike protein reduces cell hybrid during cell–cell fusion
Source: Cell Biosci. 2021 Jun 23;11:114. doi: 10.1186/s13578-021-00626-0 (PMC8220125; doi:10.1186/s13578-021-00626-0)
Supplement: Supplementary file 1 — Additional file 1: Figure S1. Quantification of fusion ratio and cell viability after cell fusion. 293T-S-WT-EGFP, 293T-S-∆19-EGFP cells were co-cultured with 293T-hACE2-mCherry cells for 24 hrs. (A) Images were taken using EVOS FL color image systems. The images were used to determine the percentage of fused cells as shown in the bar chart, there are about 34% fused cells in 293T-S-WT-EGFP co-cultured with 293T-hACE2-mCherry cells, and 24% fused cells in 293T-S-∆19-EGFP cocultured with 293T-hACE2-mCherry cells. (B) Co-culture of S-WT-293T-EGFP or S-∆19-293T-EGFP with hACE2-293T-mCherry reduced cell viability compared to related control cells. Figure S2. Increased cell size after cell fusion. Co-cultured 293T cells were harvested and fixed with 75% ethanol for 2 hour at – 20 °C, Cells were then collected and resuspended in 1 ml of PBS with RNase (at 10 µg/ml, Sigma) and propidium iodide (PI at 10 µg/ml, Sigma) for 30 min. PI stained cells were then analyzed using BD flow cytometer. DNA content was gated and analyzed using the multicycle program to determine the proportions of cell cycle and polyploidy. Co-culture of 293T-S-∆19-EGFP with 293T-hACE2-mCherry slightly increased cell size, compared to control groups. Figure S3. Spike protein mediates cell fusion in transduced SK-Hep1. Immunofluorescent images of syncytial formation when co-culture S-WT- SK-Hep1 with hACE2-Sk-Hep1 cells were obtained by confocal microscope. Figure S4. Quantification of double positive cells after cell fusion. A549-SWT-EGFP cells were cocultured with A549-hACE2-mCherry cells and analyzed by flow cytometry for mCherry and GFP co-expression at 0 hour (left) and after 24 hour (right). The three highlighted quadrants correspond to A549 cells that are either hACE2 single-positive (top left), wild-type spike protein single-positive (bottom right), or hACE2 and Spike protein double-positive (top right). Relative percentages of total live cells are displayed. The percentage of Spike-EGFP cells wer [file 13578_2021_626_MOESM1_ESM.pptx]

## Slide 1
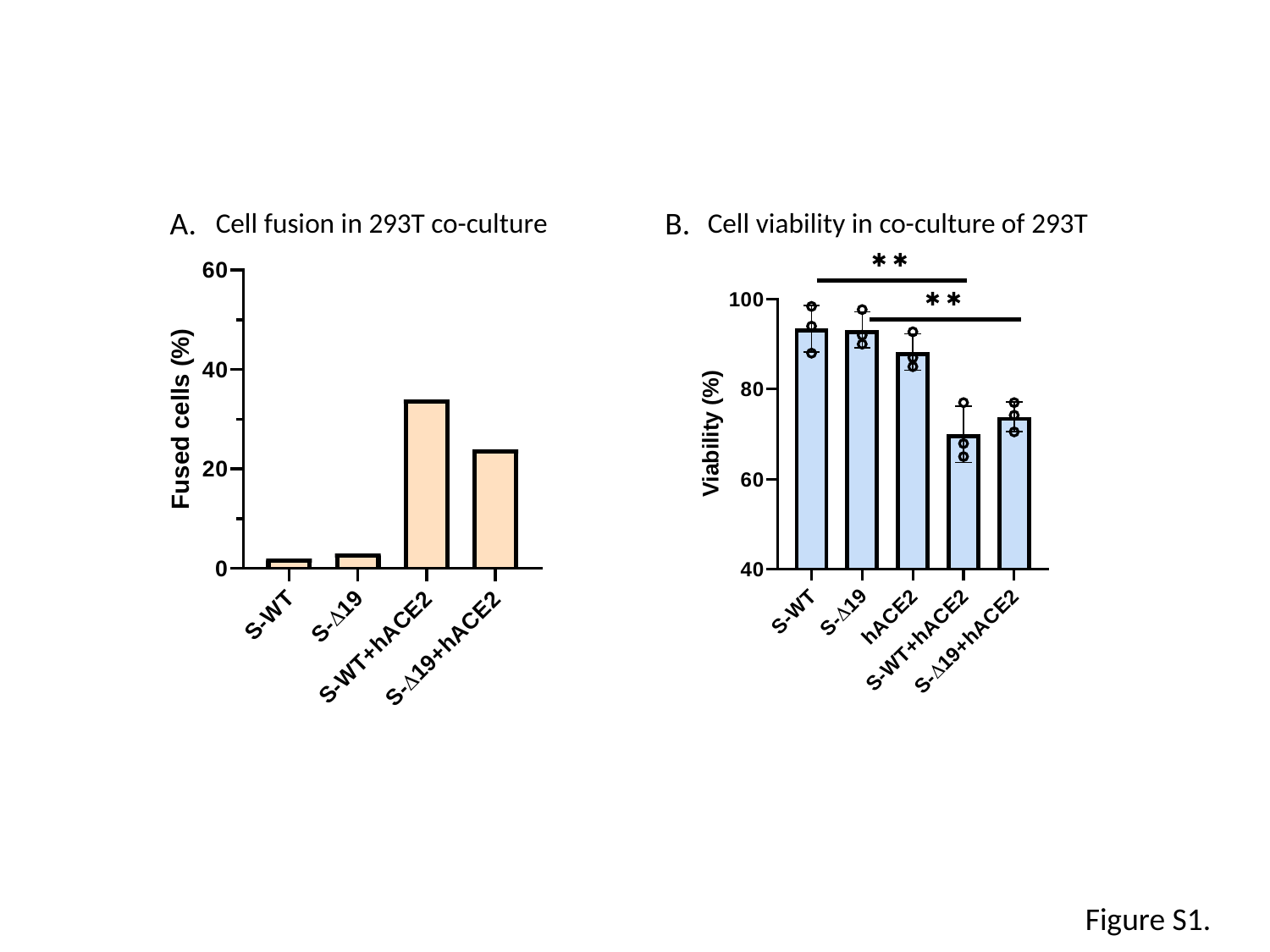

A.
B.
Cell fusion in 293T co-culture
Cell viability in co-culture of 293T
Figure S1.

## Slide 2
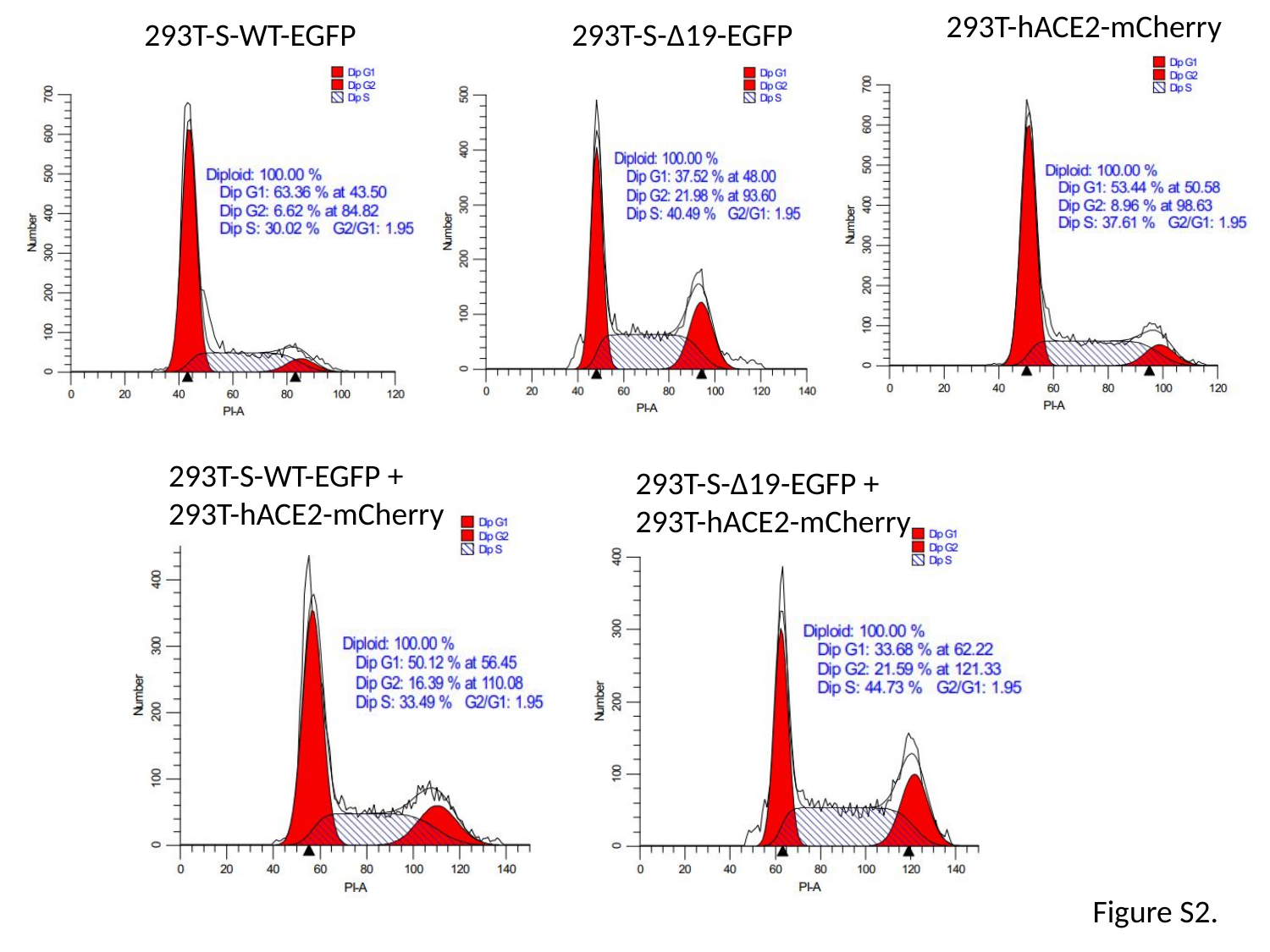

293T-hACE2-mCherry
293T-S-WT-EGFP
293T-S-∆19-EGFP
293T-S-WT-EGFP +
293T-hACE2-mCherry
293T-S-∆19-EGFP +
293T-hACE2-mCherry
Figure S2.

## Slide 3
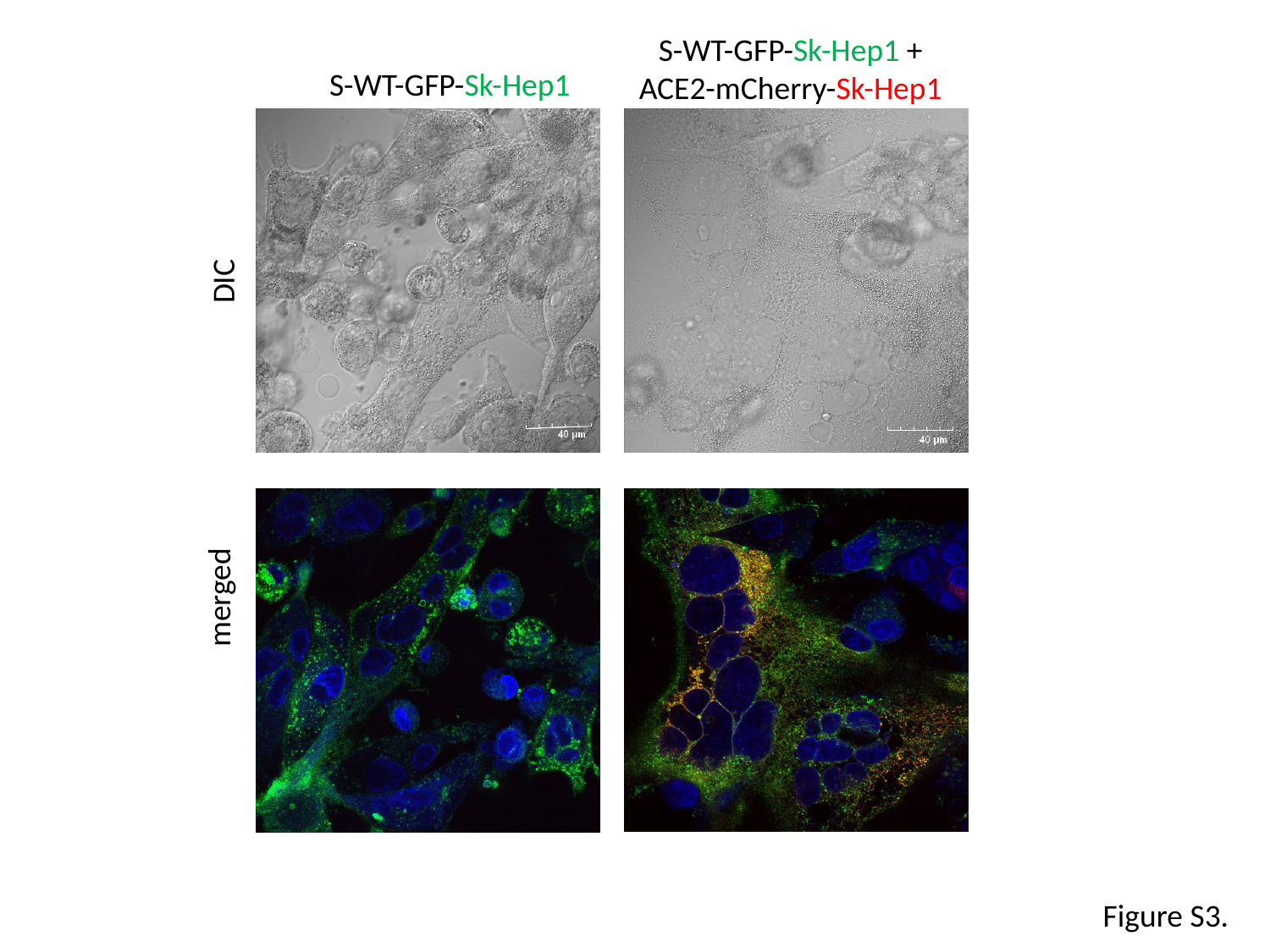

S-WT-GFP-Sk-Hep1 +
ACE2-mCherry-Sk-Hep1
S-WT-GFP-Sk-Hep1
DIC
merged
Figure S3.

## Slide 4
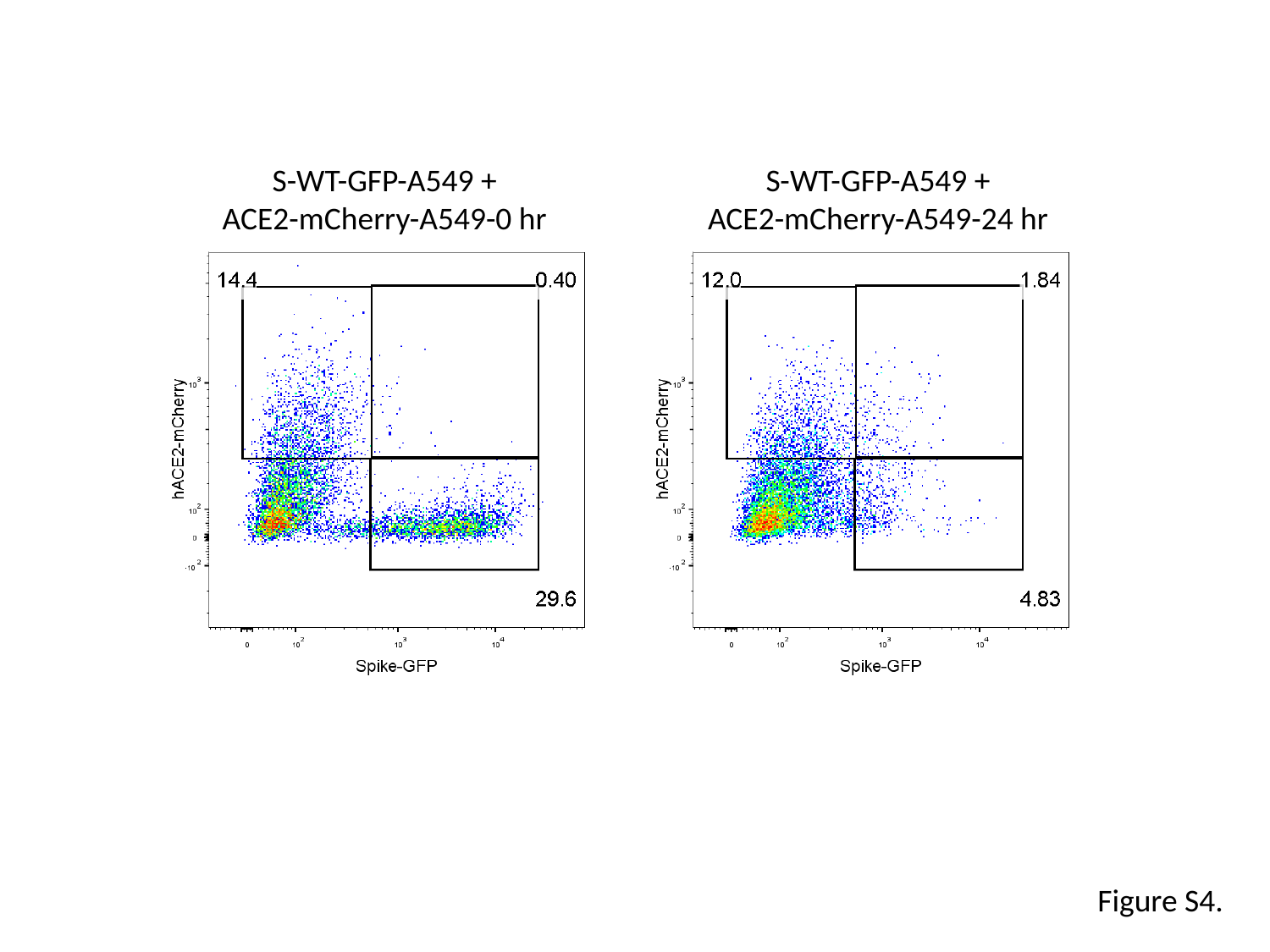

S-WT-GFP-A549 +
ACE2-mCherry-A549-24 hr
S-WT-GFP-A549 +
ACE2-mCherry-A549-0 hr
Figure S4.
